# IT WAS TERRIBLE, I DIDN'T SLEEP FOR TWO YEARS: A MIXED-METHODS EXPLORATION OF SLEEP AMONG HOSPICE FAMILY CAREGIVERS

**DOI:** 10.1093/geroni/igac059.1149

**Published:** 2022-12-20

**Authors:** Lauren Starr, Karla Washington, Miranda McPhillips, George Demiris, Debra Parker Oliver

**Affiliations:** University of Pennsylvania, Philadelphia, Pennsylvania, United States; Washington University in St. Louis, St. Louis, Missouri, United States; University of Pennsylvania, Philadelphia, Pennsylvania, United States; University of Pennsylvania, Philadelphia, Pennsylvania, United States; Washington University in St. Louis, St. Louis, Missouri, United States

## Abstract

Due to overnight caregiving demands and inadequate social support, over 1.2 million hospice family caregivers in the U.S. and millions more worldwide are at risk of poor sleep and resulting negative effects on health. Sleep problems are a modifiable source of global health inequities. The purpose of this study was to describe hospice family caregiver sleep experiences, efforts to improve sleep, and effects of sleep. Developed using the Symptom Management Model, this mixed methods study featured a concurrent nested design prioritizing qualitative reflexive thematic analysis. In 2021, 47 family caregivers of hospice care-recipients from two randomized clinical trials in the United States (NCT03712410, NCT02929108) were interviewed. Sleep-related questions from PHQ-9 and GAD-7, and self-rated health and energy were administered at baseline. Three themes emerged: quality of sleep, factors influencing sleep, and effects of sleep. Hospice family caregivers commonly experienced “interrupted” sleep with frequent night-waking due to “on-call” “vigilance” and anxiety overnight and, sometimes, in bereavement. Negative effects included exhaustion, mental and physical health decline, and reduced performance. 72.5% described sleep quality as “fair” or “poor.” At baseline, 35.5% of caregivers were bothered by trouble falling asleep, staying asleep, or sleeping too much at least “more than half the days” in a week. Caregivers were reluctant to take sleep medications. Over half quit jobs or reduced work hours to provide care; few reported adequate support. Hospice family caregivers commonly experience disordered sleep with negative effects. Clinicians must assess sleep, offer tailored sleep interventions, and provide more supports to hospice family caregivers.

